# Promoting Extracellular Electron Transfer of *Shewanella oneidensis* MR-1 by Optimizing the Periplasmic Cytochrome *c* Network

**DOI:** 10.3389/fmicb.2021.727709

**Published:** 2021-10-05

**Authors:** Weining Sun, Zhufan Lin, Qingzi Yu, Shaoan Cheng, Haichun Gao

**Affiliations:** ^1^Institute of Microbiology and College of Life Sciences, Zhejiang University, Hangzhou, China; ^2^Department of Energy Engineering, State Key Laboratory of Clean Energy, Zhejiang University, Hangzhou, China

**Keywords:** cytochrome *c*, EET, *Shewanella*, MFC, genetic engineering

## Abstract

The low efficiency of extracellular electron transfer (EET) is a major bottleneck for *Shewanella oneidensis* MR-1 acting as an electroactive biocatalyst in bioelectrochemical systems. Although it is well established that a periplasmic *c*-type cytochrome (*c*-Cyt) network plays a critical role in regulating EET efficiency, the understanding of the network in terms of structure and electron transfer activity is obscure and partial. In this work, we attempted to systematically investigate the impacts of the network components on EET in their absence and overproduction individually in microbial fuel cell (MFC). We found that overexpression of *c*-Cyt CctA leads to accelerated electron transfer between CymA and the Mtr system, which function as the primary quinol oxidase and the outer-membrane (OM) electron hub in EET. In contrast, NapB, FccA, and TsdB in excess severely impaired EET, reducing EET capacity in MFC by more than 50%. Based on the results from both strategies, a series of engineered strains lacking FccA, NapB, and TsdB in combination while overproducing CctA were tested for a maximally optimized *c*-Cyt network. A strain depleted of all NapB, FccA, and TsdB with CctA overproduction achieved the highest maximum power density in MFCs (436.5 mW/m^2^), ∼3.62-fold higher than that of wild type (WT). By revealing that optimization of periplasmic *c*-Cyt composition is a practical strategy for improving EET efficiency, our work underscores the importance in understanding physiological and electrochemical characteristics of *c*-Cyts involved in EET.

## Introduction

Electroactive bacteria capable of extracellular electron transfer (EET) have shown great potential in acting as an electroactive biocatalyst in environmentally friendly bioelectrochemical systems, such as microbial fuel cells (MFCs) and electrolysis cells (Logan and Rabaey, 2012; Shi et al., 2016). MFC, extensively studied in recent decades, is designed to be a green and sustainable technology for sustainable biodegradation of organics in wastewater and bioelectricity energy generation (Xie et al., 2013).

**FIGURE 1:**
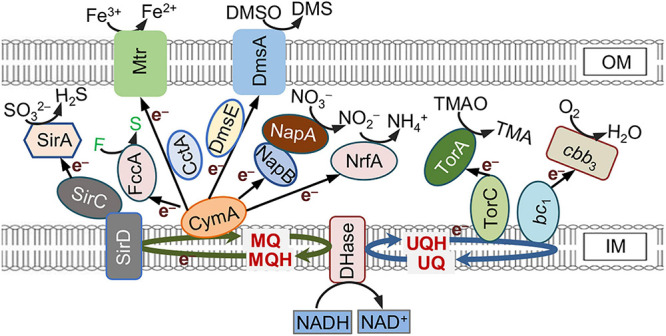
*c*-Type cytochrome (c-Cyt)-involved extracellular electron transfer (EET) pathways in *Shewanella oneidensis*. Electrons generated from oxidation of electron donors are conserved in NADH and fed into the quinol pool composed of menaquinone (MQ) and ubiquinone (UQ), which link to different quinol oxidases. CymA, the predominant quinol oxidase for EET *via* Mtr, is promiscuous for electron acceptors (EAs). This promiscuity is a likely cause of low EET efficiency. Proteins whose names are in black are *c*-Cyts whereas in white are not. F, fumarate; S, succinate.

*Shewanella oneidensis* MR-1, a γ-proteobacterial facultative anaerobe renowned for respiratory versatility, is one of the most extensively studied exoelectrogens (Hau and Gralnick, 2007). It has long been understood that a large repertoire of *c*-type cytochromes (*c*-Cyts) encoded and expressed in MR-1 serve as the molecular foundation of EET (Saffarini et al., 2003; Shi et al., 2016). Electrons derived from the oxidation of electron donors like lactate are transferred to the inner-membrane (IM) quinol pool, where they branched out into a periplasmic *c*-Cyt network through several IM-anchored quinol oxidases, including *c*-Cyts CymA (tetraheme), *bc*_1_ complex (*c*-Cyt component PetC, monoheme), TorC (pentaheme), and non-heme iron proteins such as SirD (Gon et al., 2001; Cordova et al., 2011; Marritt et al., 2012; Fu et al., 2014; Figure 1). The periplasmic network is composed of a large number of *c*-Cyts, which may function as terminal reductases only (i.e., pentaheme nitrite reductase NrfA), electron carriers only [i.e., CctA (also STC)], or both (i.e., fumarate reductase FccA) (Saffarini et al., 2003; Gao et al., 2009; Fonseca et al., 2013). This network mediates electron transport from the quinol oxidases to the Mtr system, an outer-membrane (OM) *c*-Cyt metal-reducing complex consisting of MtrCAB and OmcA (Shi et al., 2016). Mtr system is responsible for reduction of extracellular electron acceptors (EAs), such as insoluble metal oxides and electrodes, *via* EET either by the direct interaction between Mtr on the cell surface and EAs or by the indirect approach mediated by flavin electron shuttles (Kotloski and Gralnick, 2013).

The low EET efficiency of MR-1 is a major bottleneck for MFC performance, which restricts its practical applications in bioelectrochemical systems and bioremediation. Efforts have been made to improve EET efficiency and optimize the MFC performance by both genetic and engineering approaches (Li et al., 2018; Lin et al., 2018; Wang et al., 2020). In addition to advances in the chemically modified electrode, operation parameters, and bioelectrochemical reactor designs, many genetic engineering strategies, aiming at increasing the amount of releasable electrons and secreted flavin shuttles, promoting cell attachment to the anode and biofilm formation, and facilitating released electrons transferring, have been developed in recent years (Liu et al., 2015; Min et al., 2017; Li et al., 2018; Lin et al., 2018).

Given the essentiality and importance of diverse *c*-Cyts in EET of MR-1, some of these proteins have been extensively studied with respect to both physiological roles and potential targets for EET efficiency improvement. Increased electron transfer rates have been observed when the critical *c*-Cyt components of EET, such as the Mtr system and the major periplasmic electron carriers, especially CctA, are overproduced (Min et al., 2017). In particular, by replacing with CctA a few terminal reductases, including NrfA, FccA, and NapA (periplasmic nitrate reductase), Delgado et al. (2019) observed significantly increased ferric iron reduction rate and current generation. In addition, enhanced production of a large portion of *c*-Cyts by elevated cAMP levels, the effector molecule for Crp which is the primary transcriptional activator for anaerobic respiration, has also been found to be effective (Kasai et al., 2019; Cheng et al., 2020).

However, there have been reports revealing a more complex picture about effects of *c*-Cyts on EET, in the context of either the overall content or individual ones. The transport of electrons from quinol oxidases to the Mtr system is primarily mediated by CctA and FccA together, which are among the most abundant periplasmic *c*-Cyts (Fonseca et al., 2013; Alves et al., 2015; Sturm et al., 2015). However, when in excess, FccA has been reported to impair EET by altering the direction of catalysis and electron transfer through CymA-FccA interaction (McMillan et al., 2013; Jin et al., 2016a). Moreover, the electron transfer efficiency of the periplasmic *c*-Cyt network appears to be also affected by changes in the abundance of other soluble *c*-Cyts in MR-1 (Sturm et al., 2015; Jin et al., 2016a).

Although attempts to improve EET efficiency and optimize the MFCs by engineering individual and/or a portion of *c*-Cyts are many, how the composition and overall content of *c*-Cyts impact EET have not been systematically investigated. In this study, we utilized two strategies to identify *c*-Cyts in the periplasm that are deeply associated with EET efficiency in MFCs. On one hand, by constructing mutants devoid of each of the soluble *c*-Cyts, its capacity in transferring electrons from CymA to the Mtr complex was assessed. On the other hand, we overproduced these *c*-Cyts individually in the wild type (WT) to pinpoint those that dissipate electrons obtained from the quinol pool. Then, based on the results from both strategies, engineered strains were constructed in all possible combinations and their EET efficiency was characterized by bioelectricity generation. Our data revealed that a strain lacking FccA, NapB, and TsdB while overproducing CctA achieved the highest maximum power density. Furthermore, the EET efficiency of this strain can be further improved with addition of flavin.

## Materials and Methods

### Bacterial Strains, Plasmids, and Culture Conditions

A list of all bacterial strains and plasmids used in this study is given in Table 1. All chemicals were acquired from Sigma-Aldrich Co., (Shanghai, China) unless otherwise noted. For genetic manipulation, *Escherichia coli* and *S. oneidensis* strains under aerobic conditions were grown in Lysogeny Broth (LB, Difco Laboratories Inc., Detroit, MI, United States) Lennox, at 37 and 30°C, respectively. When needed, the growth media were supplemented with chemicals at the following concentrations: 2,6-diaminopimelic acid (DAP), 0.3 mM; ampicillin sodium, 50 μg/ml; kanamycin sulfate, 50 μg/ml; and gentamycin sulfate, 15 μg/ml.

**TABLE 1:** Strains and plasmids used in this study.

**Strain or plasmid**	**Description**	**References or source**
***Escherichia coli* strains**		
DH5α	Host for cloning	Laboratory stock
WM3064	Δ*dapA*, donor strain for conjugation	W. Metcalf, UIUC
***Shewanella oneidensis* strains**		
MR-1	Wild type	Laboratory stock
HG2727	Δ*cctA* derived from MR-1	This study
HG0970	Δ*fccA* derived from MR-1	Gao et al. (2010)
HG 0845	Δ*napB* derived from MR-1	Jin et al. (2016b)
HG4048	Δ*tsdB* derived from MR-1	This study
HG4485	Δ*SO4485* derived from MR-1	This study
HG0845-0970	Δ*napB*Δ*fccA* derived from MR-1	This study
HG0970-4048	Δ*fccA*Δ*tsdB* derived from MR-1	This study
HG0845-4048	Δ*napB*Δ*tsdB* derived from MR-1	This study
HG0845-0970-4048	Δ*napB*Δ*fccA tsdB* derived from MR-1	This study
HG0608-0610-SO0483-0484	Δ*petABC*Δ*sirCD* derived from MR-1	This study
**Plasmid**		
pHGE-Ptac	Km^*r*^, IPTG-inducible expression vector	Luo et al. (2013)
pHGEN-Ptac-ccmFGH	P*tac*-*ccmFGH* within pHGEN-Ptac	This study
pHGEN-Ptac-cctA	P*tac*-*cctA* within pHGEN-Ptac	This study
pHGEN-Ptac-fccA	P*tac*-*fccA* within pHGEN-Ptac	Jin et al. (2016a)
pHGEN-Ptac-tsdB	P*tac*-*tsdB* within pHGEN-Ptac	This study
pHGEN-Ptac-napB	P*tac*-*napB* within pHGEN-Ptac	Jin et al. (2016b)
pHGEN-Ptac-scyA	P*tac*-*scyA* within pHGEN-Ptac	Yin et al. (2015)
pHGEN-Ptac-SO3300	P*tac*-*SO3300* within pHGEN-Ptac	This study
pHGEN-Ptac-SO3056	P*tac*-*SO3056* within pHGEN-Ptac	This study
pHGEN-Ptac-SO1413	P*tac*-*SO1413* within pHGEN-Ptac	This study
pHGEN-Ptac-SO4485	P*tac*-*SO4485* within pHGEN-Ptac	This study
pHGEN-Ptac-SO4484	P*tac*-*SO4484* within pHGEN-Ptac	This study
pHGEN-Ptac-SO4142	P*tac*-*SO4142* within pHGEN-Ptac	This study
pHGEN-Ptac-SO4666	P*tac*-*SO4666* within pHGEN-Ptac	This study
pHGEN-Ptac-SO717	P*tac*-*SO0717* within pHGEN-Ptac	This study
pHGEN-Ptac-SO714	P*tac*-*SO0714* within pHGEN-Ptac	This study
pHGEN-Ptac-SO3420	P*tac*-*SO3420* within pHGEN-Ptac	This study

### Mutant Construction and Complementation

In-frame deletion strains for *S. oneidensis* were constructed using the *att*-based fusion PCR method as described previously (Jin et al., 2013). In brief, two fragments flanking the target gene were amplified by PCR with outside primer containing *attB* and the gene-specific sequence and inside primer containing the linker and the gene-specific sequence, which were joined by the second round of PCR with two outside primers. The fusion fragments were introduced into plasmid pHGM01 by using Gateway BP clonase II enzyme mix (Invitrogen) according to the manufacturer’s instruction, resulting in mutagenesis vectors, which were maintained in *E. coli* DAP auxotroph WM3064. The vectors were subsequently transferred into relevant *S. oneidensis* strains *via* conjugation. Integration of the mutagenesis constructs into the chromosome was selected by resistance to gentamycin and confirmed by PCR. Verified transconjugants were grown in LB broth in the absence of NaCl and plated on LB containing 10% sucrose. Gentamycin-sensitive and sucrose-resistant colonies were screened by PCR for deletion of the target gene. Mutants were verified by sequencing the mutated regions.

Growth of *S. oneidensis* mutant strains generated in this study was measured by recording the optical density at 600 nm (OD_600_) values in triplicate with the wild type as the control in MS minimal medium with 30 mM L-sodium lactate (Shi et al., 2015). For genetic complementation of the mutants and inducible gene expression, genes of interest generated by PCR were placed under the control of isopropyl β-D-1-thiogalactoside (IPTG)-inducible promoter P*tac* within pHGE-P*tac* (Luo et al., 2013). After verification by sequencing, the resultant vectors in *E. coli* DAP auxotroph WM3064 were transferred into the relevant strains *via* conjugation. Throughout this study, when compared with strains expressing *c*-Cyt genes from plasmid pHGE-Ptac in the presence of required antibiotics, MR-1 (the WT) carrying the empty vector was used unless otherwise noted.

### *c*-Type Cytochrome Assays

Unless otherwise noted, cultures of the early stationary phase were used for chemical assays. Cyt *c* abundance of strains was first estimated by the color intensity of cell pellets. For quantification, cells were collected by centrifugation and suspended in PBS buffer. The OD_600_ of each cell suspension was adjusted to 1 and lysed, and total heme in 50 μl of cell suspension aliquot for each strain was determined with the QuantiChrom heme assay kit (BioAssay Systems, Hayward, CA, United States) according to the manufacturer’s instructions. The bound heme *c* (*c*-Cyt) levels for each strain were obtained by subtracting the average values of the *c*-Cyt-deficient Δ*ccmF* strain. The relative abundance of heme *c* was then calculated normalizing to the average heme *c* values of the WT.

### Microbial Fuel Cell Setup

A dual-chambered MFC (total working volume is 140 ml) was used to evaluate EET efficiency of *S. oneidensis* strains. Two chambers were separated by Nafion 117 membrane, which was pretreated with 3% hydrogen peroxide, distilled water, 0.5 M sulfuric acid, and then distilled water for 1 h under 80°C. Carbon cloth was used as electrode materials for both anode (1 cm × 1 cm) and cathode (1.5 cm × 2.0 cm). The anodic electrolyte was composed of 5% LB-Lennox and 95% M9 buffer with 20 mM lactate as electron donor (Li et al., 2018). The cathodic electrolyte was made of 50 mM K_3_[Fe(CN)_6_], 50 mM KH_2_PO_4_, and 50 mM K_2_HPO_4_. Fresh medium was inoculated with overnight cultures to an OD_600_ of ∼0.01 and shaken at 200 rpm at 30°C. After 12 h, cells were collected, washed three times with anodic electrolyte, and resuspended with anodic electrolyte containing kanamycin and IPTG at desirable concentrations to an OD_600_ of ∼0.7. To maintain anaerobic conditions, the anodes of MFC were purged with N_2_ gas. The anode and cathode were connected with wires and an external resistor (2 kΩ). The output voltages across the external resistor were recorded at a rate of 1 point per 30 min using a data acquisition system (Keysight 34970A). The MFCs were operated at 30°C. All measurements were conducted with at least three biological replicates (cells in independently operated MFCs).

### Bioelectrochemical Analyses

The polarization curves and power density curves of MFCs were determined by measuring the stable output voltage generated across various external resistances (50–100,000 Ω). Current was calculated using the equation, *I* = *V*/*R*, where *I* is the current, *V* is the measured cell voltage, and *R* is the applied resistance. Power was calculated using *P* = *IV*. Both current and power densities were normalized to anode surface area. Cyclic voltammetry (CV) was performed using an electrochemical work station (CHI600E; Chenhua, Shanghai, China) in a three-electrode configuration with Ag/AgCl electrode (+ 0.199 V vs. SHE) as a reference electrode to evaluate the efficiency of the electron transfer from the electrode to bacterial cells. The electrolyte was continuously stirred by a magnetic stirrer during CV scanning. CV sweeps were performed from −0.6 to 0.2 V (vs. Ag/AgCl), and scan rate was 1 mV/s. Electrochemical impedance spectroscopy (EIS) experiments were used to evaluate the internal resistance of the MFCs over a frequency range from 0.01 Hz to 100 kHz at an open circuit potential with an amplitude signal of 5 mV.

### Biofilm Characterization

Biofilms attached to the anode carbon cloth were observed by scanning electron microscope (SEM, S-4800, Hitachi, Tokyo, Japan). The samples were prepared essentially the same as described elsewhere (Liu et al., 2015). The anode carbon cloth was collected at the specified time point, and it was dipped in 1% glutaraldehyde overnight. Samples were washed with phosphate-buffered saline (PBS, pH 7.4) three times. Then they were dehydrated with gradient concentrations of ethanol solutions (30, 50, 70, 80, 90, and 100%). and then vacuum dried. Samples were coated with Au prior to the SEM imaging.

Bicinchoninic acid (BCA) measurement was conducted to detect the total biomass of the biofilms grown on the anodes. The anode carbon cloth was washed with 2 ml PBS to remove bacterial cells attached on it. Then this washing-off buffer was sonicated for 30 s on ice to fully lyse cells. The protein content of bacterial suspension was determined by the BCA protein assay kit according to the manufacturer’s instruction.

### Methyl Orange Degradation Tests

The recombinant and the control strains were cultured in LB-Lennox medium at 30°C. After 12 h cultivation, cells were collected, washed with PBS for three times, and resuspended in MS mineral medium (Shi et al., 2015). Twenty-millimolar lactate and 30 mg/L methyl orange (MO) were added into MS and used as the sole carbon source and the electron acceptor, respectively. The medium in each serum vial was sparged with N_2_ to ensure an anaerobic atmosphere. At each time point, the samples were taken by syringe and immediately applied to MO concentration determination with a Synergy 2 multimode microplate reader (M200 Pro, Tecan) at 465 nm. Standard curves were made with a series of MO dilutions each time.

### Other Analyses

Student’s *t*-test was performed for pairwise comparisons. When necessary, values are presented as means ± standard deviation (SD) in figures.

## Results

### Elevation in the Overall *c*-Type Cytochrome Content of MR-1 Does Not Improve Bioelectricity Generation in Microbial Fuel Cell

**FIGURE 2:**
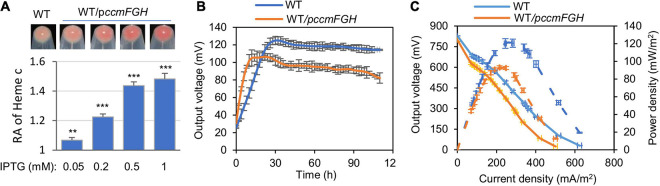
Physical and bioelectrochemical characterization of MR-1 overproducing CcmFGH. **(A)** Cell pellet color and levels of heme *c* of indicated strains grown anaerobically on TMAO. Cells in late-exponential phase cultures were pelleted, photographed, and then lysed for quantification of heme *c* levels. The average heme *c* level in MR-1 [the wild type (WT)] was set to 1, to which the heme *c* levels in other strains were normalized, giving the relative abundance (RA). p*ccmFGH*, controlled expression within plasmid pHGE-Ptac under the control of isopropyl β-D-1-thiogalactoside (IPTG)-inducible promoter P*tac*. **(B)** Output voltage of microbial fuel cells (MFCs) incubated with indicated recombinant and the control (WT, carrying empty vector) strains across the 2,000 Ω external resistor. **(C)** Polarization curves and power density curves determined by measuring the stable output voltage generated across various external resistances. In panels **(B,C)**, IPTG, 0.5 mM. Three independent operations (biological replicates, throughout the study) were performed, and data are presented by the means ± standard deviation (SD). In panel **(A)**, asterisks indicate statistically significant difference when compared with the WT values (**p < 0.01; ***p < 0.001). In both **(B,C)**, the differences in the peak values between two strains were <0.05.

It is well recognized that the molecular foundation for EET in bacteria is *c*-Cyts (Shi et al., 2016; Lovley and Walker, 2019; Wang F. et al., 2019). To promote cell current generation of MR-1, we intended to increase the overall *c*-Cyt abundance in a proportional manner. In accordance with an unusually large repertoire of *c*-Cyts, MR-1 is equipped with a robust cyt *c* maturation system characterized by three functional modules encoded by three operons, respectively, *ccmABCDE* (heme transport), *ccmI* (complexion), and *ccmFGH* (heme-apocyt *c* ligation) (Jin et al., 2013; Fu et al., 2015). Given that the CcmFGH module alone (in the absence of CcmABCDE), when overexpressed, supports *c*-Cyt maturation (San Francisco et al., 2014), we reasoned that overexpressing *ccmFGH* in the WT background would enhance *c*-Cyt production. Indeed, expression of an additional copy of *ccmFGH* driven by IPTG-inducible promoter P*tac* with 0.05 mM IPTG and above enabled WT (WT/p*ccmFGH*, grown on TMAO, an EA that supports growth best) to produce more *c*-Cyts, which can be readily judged by deepened reddish-brown color of cell pellets, a signature feature endowed by *c*-Cyts (Figure 2A). In the presence of 1 mM IPTG, a ∼40% increase in the overall quantity of *c*-Cyts was achieved.

To investigate impacts of *c*-Cyt overproduction on bioelectrochemical capability, MFCs incubated with the WT expressing *ccmFGH* or not were set up and operated under batch mode. To our surprise, although WT/p*ccmFGH* with elevated *c*-Cyt abundance (grown with 0.5 mM IPTG) initiated EET more promptly, the maximum output voltage (∼106.4 mV) that it produced was significantly lower than the WT did (∼125.1 mV), based on the output voltage vs. time curves (Figure 2B). Similar results were obtained from the polarization curves (output voltage vs. current density) and power density curves (power density vs. current density) of MFCs (Figure 2C). The potential dropped in output voltage vs. current density curves along with the increase in current density by reducing the external load resistance. A higher slope of MFCs inoculated with the WT containing overabundant *c*-Cyts indicates an increase in internal ohmic resistance compared with that of the control strain (Figure 2C). The maximum power density of the MFCs inoculated with the WT was 120.5 mW/m^2^, significantly higher than that with the high *c*-Cyt content (93.1 mW/m^2^). The maximum power density of the WT was cut by about 30% after *ccmFGH* genes were overexpressed in it. These data rule out the possibility that the EET activity of MR-1 increases with the total *c*-Cyt content.

### Some Periplasmic *c*-Type Cytochromes Impact Extracellular Electron Transfer Efficiency

The most likely explanation for this unexpected finding would be that certain *c*-Cyts, when in excess, interfere with EET efficiency. Coincidently, although the EET of MR-1 is composed of only several *c*-Cyts (Min et al., 2017; Delgado et al., 2019), earlier studies revealed that many *c*-Cyts significantly influence the EET efficiency in their absence and overproduction (Bretschger et al., 2007; Gao et al., 2010). In MR-1, it is firmly established that EET critically relied on the quinol dehydrogenase CymA at the IM and the Mtr system at the OM (Kracke et al., 2015; Shi et al., 2016). To bridge CymA and Mtr, cells utilize a redox network composed of periplasmic *c*-Cyts, which has been proposed to be the bottleneck of EET efficiency (Fonseca et al., 2013).

**FIGURE 3:**
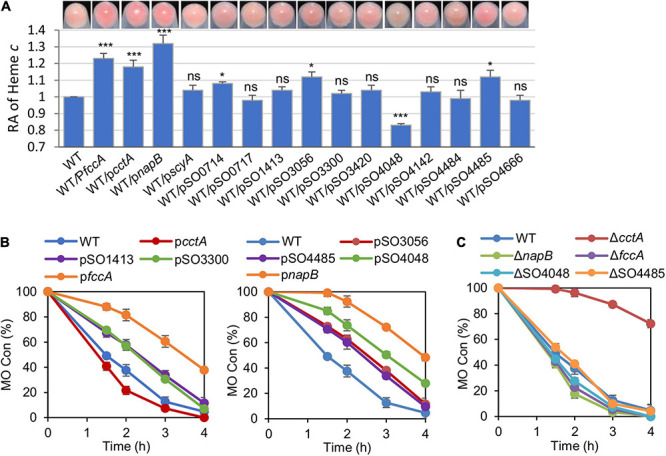
The periplasmic *c*-Cyts in overabundance interfere with EET efficiency. **(A)** Cell pellet color and levels of heme *c* of indicated strains grown anaerobically on TMAO with 0.5 mM IPTG. Data were processed and presented as described in Figure 2A. **(B)** Methyl orange (MO) kinetic traces of the indicated strains grown with 0.5 mM IPTG. **(C)** MO kinetic traces of indicated periplasmic *c*-Cyt single mutants grown with 0.5 mM IPTG. Three independent operations were performed, and data are presented by the means ± SD. In panel **(A)**, asterisks indicate statistically significant difference when compared with the WT values (ns, not significant; *p < 0.05; ***p < 0.001). In both **(B,C)**, the differences in the peak values between two strains were <0.05.

Among 42 *c*-Cyt proteins of MR-1, 27 were predicted to be located in the periplasm but only approximately half of them have been detected in proteomics studies (Meyer et al., 2004; Nissen et al., 2012). By combining proteomics data and the general fact that *c*-Cyts of low molecular mass function primarily as electron carriers, we chose 14 *c*-Cyt proteins with a molecular mass less than 22 kDa and verified electron transfer protein FccA in this study (Supplementary Table 1). To assess whether these *c*-Cyts interfere with EET efficiency when overproduced, we manipulated their production by expressing them by IPTG-inducible promoter P*tac* within vectors pHGE-Ptac. Based on the cell-pellet colors, it was immediately evident that some of periplasmic *c*-Cyts in excess (with 0.5 mM IPTG), impacted the overall *c*-Cyt content significantly (Figure 3A). While cells overproducing CctA, FccA, and NapB elevated the overall *c*-Cyt content by at 20% and more, SO4048 in excess exhibited a negative effect, by approximately 16%. To quickly assess the EET capacity of these strains, reduction of MO was performed. MO, an azo compound, can be extracellularly reduced by MR-1 in an EET-dependent manner (Min et al., 2017). As shown in Figure 3B (as well as in Supplementary Figures 1A,B), only the strain overproducing CctA had improved MO reduction whereas 7 strains (overexpressing FccA, NapB, SO1413, SO3056, SO3300, SO4485, and SO4048) had compromised ability to reduce MO. This phenomenon, which is in line with the results of CcmFGH overproduction, suggest that the total *c*-Cyt contents are not critically linked to electron transfer capacity of MR-1 strains, and *c*-Cyts have to be characterized individually and specifically for their role in EET.

We then examined the impacts of the depletion of these proteins on the *c*-Cyt content and MO reduction. Removing any of these periplasmic *c*-Cyts did not significantly affect the overall *c*-Cyt content (data not shown). Despite this, although most of these strains were not distinguishable from the WT in MO reduction, those lacking CctA, FccA, NapB, or SO4048 exhibited significant difference (Figure 3C and Supplementary Figures 1C,D). The *cctA* mutant showed a substantially reduced reduction rate, but the loss of FccA, NapB, or SO4048 improved the reduction. All of these data, collectively, suggest that the redox network bridging CymA and the Mtr system is likely composed of many periplasmic *c*-Cyts, and the efficiency of the network requires coordinated production of all participants.

### The Contributions of Periplasm *c*-Type Cytochromes in Extracellular Electron Transfer Efficiency Vary

As shown above, the enhanced current-generating capacity was observed from strains depleted of three periplasmic *c*-Cyts, FccA, NapB, and SO4048. In MR-1, FccA and NapB function as fumarate reductase and the electron transfer subunit of nitrate reductase, respectively, and their involvements in transferring electrons from CymA to the Mtr system have been confirmed (Gao et al., 2009; Jin et al., 2016b). SO4048, now named TsdB, is highly likely to be TsdB (therefore named TsdB), the electron transfer subunit of thiosulfate oxidase, given its high sequence conservation to TsdB of Sideroxydans lithotrophicus (BLASTp e-value, 2e-40) (Kurth et al., 2016).

**FIGURE 4:**
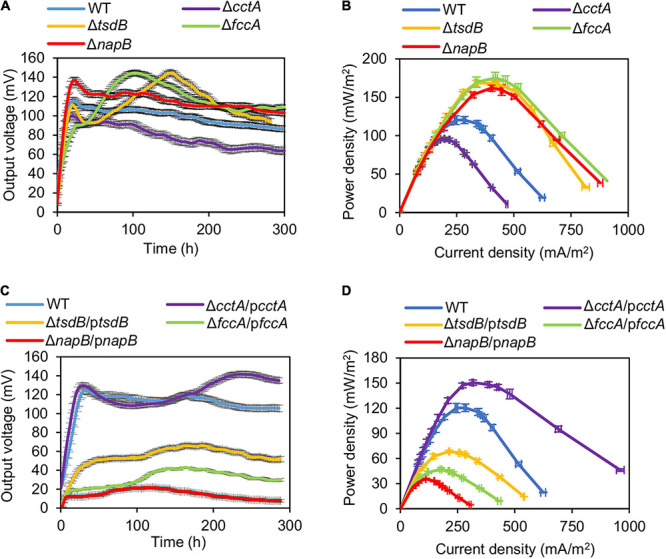
Bioelectrochemical characterization of periplasmic *c*-Cyt mutants in dual-chamber MFCs. Data from WT and indicated periplasmic *c*-Cyt mutants w/o complementation with 0.5 mM IPTG are shown. **(A,C)** Output voltage of MFCs incubated with the indicated strains across the 2,000 Ω external resistor. **(B,D)** Power density curves determined by measuring the stable output voltage generated across various external resistances. Three independent operations were performed, and data are presented by the means ± SD. In all panels, the differences in the peak values between WT and each of periplasmic *c*-Cyt mutants under test were <0.01.

To test the electrochemical performance of the strains lacking CctA, FccA, NapB, and TsdB, the classic two-chambered MFCs were set up and operated at 30°C with an external resistance of 2,000 Ω. The output voltage vs. time profiles of the MFCs incubated with strains under test induced with IPTG at 0.1 and 0.5 mM were recorded for 300 h. The output voltage of MFCs incubated with Δ*cctA* generated a substantially lowered output voltage peak (∼98 mV), compared with that of the parental WT (120.3 mV) (Figure 4A). On the contrary, output voltage peaks of the Δ*tsdB*, Δ*fccA*, and Δ*napB* strains were significantly improved, reaching to 144.5, 143.8, and 136.6 mV, respectively (Figure 4A). Notably, these mutants had the differently output voltage dynamics: Δ*napB* resembled the WT except for higher output whereas the other two mutants took a much longer time to reach the peaks. These MFCs maintained output voltages above 70% of the maximum value for about 300 h.

For validation, the polarization and power density curves of MFCs incubated with these strains were measured. Results showed that the MFCs inoculated with the mutants Δ*tsdB*, Δ*fccA*, and Δ*napB* generated higher power density than the WT (Figure 4B). The dropping slope of these three mutants was much smaller than the WT, indicating that their internal resistance in MFC was smaller than that of the WT (Supplementary Figure 2). The maximum power densities achieved by Δ*tsdB*, Δ*fccA*, and Δ*napB* were 168.4, 169.7, and 162.1 mW/m^2^, respectively, approximately 1.40, 1.41, and 1.35 times higher than that of the WT. As, similarly expected, Δ*cctA* had a reduced maximum power density (95.9 mW/m^2^) compared with the WT, reinforcing that CctA played an important role in the outward EET of MR-1.

Impacts of these soluble *c*-Cyts on current-generating capacity were further verified with them in overabundance. Δ*cctA* overproducing CctA (Δ*cctA*/p*cctA*, with 0.5 mM IPTG; results with 0.1 mM IPTG are shown in Supplementary Figure 2) showed increased output voltage peak (141.6 mV), higher than that of the WT although the peak arrived later (Figure 4C). In line with this, the maximum power density of this strain, 150.5 mW/m^2^, was also significantly higher than that of the WT (Figure 4D). In contrast, when overproduced, TsdB, FccA, and NapB substantially compromised current-generating capacity. All of these data indicate that except for CctA, other components of the redox network bridging CymA and the Mtr system mainly negatively influence the EET efficiency.

### Proper Modification of Periplasm *c*-Type Cytochromes Improved Extracellular Electron Transfer Ability of MR-1

**FIGURE 5:**
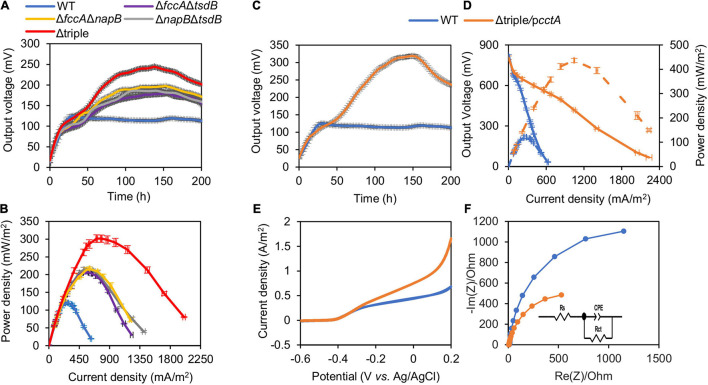
Optimized MFC performance of engineering strains. Data from mutants are given in panels **(A–F)** show triple mutants overexpressing *cctA* with 0.5 mM IPTG. **(A,C)** Output voltage of MFCs incubated with the indicated strains across the 2,000 Ω external resistor. **(B)** Power density curves determined by measuring the stable output voltage generated across various external resistances. **(D)** Polarization curves and power density curves (dashed lines) determined by measuring the stable output voltage generated across various external resistances. **(E)** Cyclic voltammetry (CV) characterization of the MFC with inoculations of the control strain and indicated recombinant strains under turnover condition, respectively. **(F)** Nyquist plots (0.01 Hz to 100 kHz at the bias potential 0.2 V vs. Ag/AgCl with a 5-mV perturbation signal of different strains in MFCs. Three independent operations were performed, and data are presented by the means ± SD. In all panels, the differences in the peak values between WT and each of mutants under test were <0.01.

As the presence of three periplasmic *c*-Cyts FccA, NapB, and TsdB compromises the EET efficiency of MR-1, engineered strains lacking their coding genes in all possible combinations were constructed. Not surprisingly, the output voltages of MFCs incubated with Δ*fccA*Δ*napB*, Δ*fccA*Δ*tsdB*, and Δ*napB*Δ*tsdB* were significantly elevated in these strains by at least 44.4 and 25.5% relative to those of the WT and any single mutant, respectively (Figure 5A). Consistently, the power density values of these double mutants increased substantially (∼70%), reaching levels higher than 200 mW/m^2^ (Figure 5B). These observations manifest that the impacts of these *c*-Cyts are additive. In line with this notion, the triple mutant, in which all *napB*, *fccA*, and *tsdB* were removed, achieved output voltage peaks and power density at further elevated levels, up to 244.3 mV and 290 mW/m^2^, respectively, which are nearly 1.95 and 2.4 times those of the WT (125.1 mV; 120.5 mW/m^2^) (Figures 5A,B).

Subsequently, we tested the effect of CctA in overabundance on the EET efficiency of the triple mutant (Δtriple/p*cctA*). When induced with 0.5 mM IPTG, the production of CctA elevated output voltage to 318.8 mV (Figure 5C). The total electrons harvested during the 200-h MFC operation were then calculated by the integration of the voltage output curves (in Figures 5A,C) divided by the external resistance at 2 kΩ. The MFC incubated with Δtriple/p*cctA* (0.5 mM IPTG) produced 80.4 coulombs of electrons in total, which was ∼2.01 times higher than that of the WT. Correspondingly, the coulombic efficiencies of Δtriple/p*cctA* and the WT were 14.58 and 7.24%, respectively. Consistently, based on the polarization and power output curves of Δtriple/p*cctA* (Figure 5D), Δtriple/p*cctA* achieved a maximum power density output of 436.5 mW/m^2^, which was significantly better than the WT (125.1 mV and 120.5 mW/m^2^).

**FIGURE 6:**
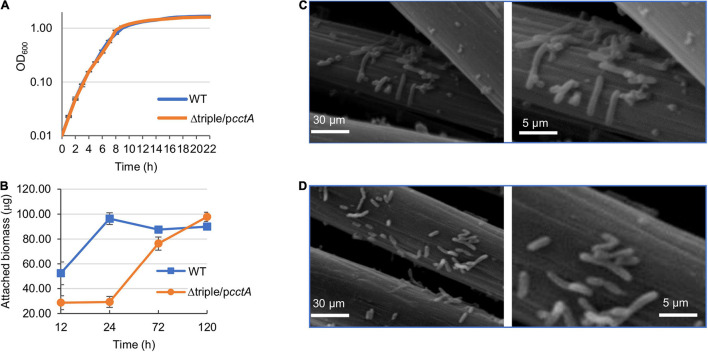
Impacts of mutations with overexpressed CctA on growth and biofilm structure. **(A)** Growth of WT and Δtriple/p*cctA* on TMAO. Cells growing exponentially were inoculated into the fresh MS with 30 mM TMAO as the EA. Growth was monitored by recording the optical density at 600 nm. **(B)** Biofilm mass of Δ*triple*/p*cctA* and WT formed on the anode of MFC. At each time point, the total biomass of the biofilms grown on the anodes was determined by using bicinchoninic acid (BCA) measurement as described in the section “Materials and Methods.” **(C,D)** Characterization of the anodic biofilms of MFC after output voltage reaching the peak. SEM images of the biofilm of WT **(C)** and Δtriple/p*cctA* (with induction of 0.5 mM IPTG) **(D)**. The right panels represent a portion of the images on the left panels enlarged. Three independent operations were performed with data presented in panels **(A,B)** by the means ± SD and representative images shown in panels **(C,D)**.

As Δtriple/p*cctA* represents the most optimized periplasmic *c*-Cyt network in MR-1, it was further characterized with respect to MFC performance. The CV scan results of Δtriple/p*cctA* and WT showed from the wave of CV curve under turnover conditions, a flavin-dependent current from −0.43 V and a higher-potential wave with an onset at −0.30 V, which reflects the oxidized electric current facilitated by direct EET through the OM *c*-Cyts (Figure 5E). Clearly, this current generated by Δtriple/p*cctA* had a peak density substantially higher than that of the WT (∼1.5 vs. ∼0.6 A/m^2^). Moreover, EIS, which reflects the internal interfacial charge transfer resistance (R*_*ct*_*) of the MFCs, was determined (Figure 5F). R*_*ct*_* is represented by the diameter of the semicircle of the Nyquist plot, and a smaller R*_*ct*_* indicates a faster electron-transfer rate. Results showed that the R*_*ct*_* value decreased remarkably from 2,560 Ω of WT to 1,163 Ω of Δtriple/p*cctA*, supporting that Δtriple/p*cctA* has a faster electron transfer. All of these data, collectively, conclude that proper modification of periplasm *c*-Cyts is able to improve EET efficiency of MR-1 by increasing electron harvest and current density, reducing the resistance of electrochemical reactions on the electrode, and accelerating electron transfer, leading to enhanced MFC performance. Subsequently, we examined whether the elevated current production by the Δtriple/p*cctA* strain is associated with growth and biofilm formation as the association of cells with electrodes is a critical factor for EET (Borole et al., 2011). When grown on TMAO, the Δtriple/p*cctA* strain was indistinguishable from the WT (Figure 6A), suggesting that the increased current production is not due to a general fitness gain. Similarly, biofilm quantification exhibited that both the strains exhibited ∼30 μg/cm^2^ protein attached on the electrodes after the output voltage reaching the peak (Figure 6B). In addition, the SEM analysis of these fully developed biofilms revealed that this engineered strain behaved, similarly, when compared with the WT, with respect to biofilm structure on the surfaces of MFC anodes (Figures 6B,C). However, it should be noticed that the Δtriple/p*cctA* strain formed the biofilm significantly slower than the WT, taking four more days to obtain the equal biomass of the WT biofilm on the anode (Figure 6B). This difference may explain that V-t curves of Δtriple/p*cctA* increased very slowly over time and reached a peak 100 h later than the WT (Figures 5A,C). Thus, the enhanced current production in Δtriple/p*cctA* is a result of intrinsic physiological change other than altered interaction between cells and electrodes.

### Exogenous Flavin Rather Than the Removal of the Additional Outlets of the Quinol Pool Elevated Extracellular Electron Transfer Rate and Electricity Power Output of the Engineered Strain

In addition to CymA, there are other proteins that could function as quinol dehydrogenases in MR-1, including the cyt *bc*_1_ complex (encoded by the *petABC* operon), SirCD, and TorC, resulting in branched electron transport pathways (Gon et al., 2001; Cordova et al., 2011; Fu et al., 2014; Kracke et al., 2015). While TorC is a Cyt-*c* that specifically mediates electron transfer from the quinol pool to TMAO reductase TorA, both the cyt *bc*_1_ complex and SirCD are coupled to multiple electron carriers, albeit much less effective than CymA (Cordova et al., 2011; Fu et al., 2014; Figure 1).

To evaluate the interference of additional electron transport branches, we deleted *petABC* and *sirCD* from the WT and assayed the resulting strain with respect to EET efficiency. The data from MFCs with an external resistance of 2,000 Ω revealed that the additional depletion of both the cyt *bc*_1_ complex and SirCD had very limited, but significant, impacts on EET efficiency (Supplementary Figure 3A). All EET characteristic values of the mutant, including maximum output voltage peak (Supplementary Figure 3A) and maximum power density (Supplementary Figure 3B), were only slightly higher than those of the WT. We then removed *petABC* and *sirCD* from Δtriple/p*cctA* but found that the additional loss of these two quinol dehydrogenases did not elicit significant differences in EET efficiency (Supplementary Figure 3C). Thus, although the removal of the additional outlets of the quinol pool has a beneficial impact on EET efficiency in the WT background, this effect can be overshadowed by the reorganization of the periplasmic electron transfer network.

**FIGURE 7:**
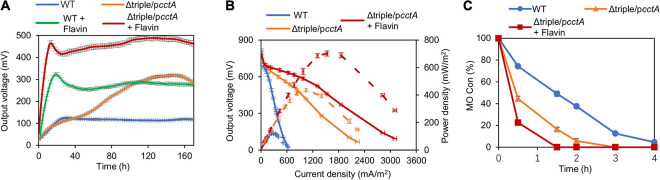
Impacts of riboflavin (RF) on MFC performance of the optimized strain. **(A)** Output voltage of MFCs incubated with the Δtriple/p*cctA* and WT, across the 2,000-Ω external resistor without or with the addition of RF. **(B)** Polarization curves and power density curves (dashed lines) determined by measuring the stable output voltage generated across various external resistances. **(C)** MO kinetic traces of the Δtriple/p*cctA* without or with addition of RF. Three independent operations were performed, and data are presented by the means ± SD. In all panels, the differences in the peak values between the strains under test without or with addition of RF were <0.01.

In addition to the electron transport by direct contact, electron-shuttle compounds, such as flavin mononucleotide (FMN) and riboflavin (RF), synthesized and secreted by MR-1 or added exogenously, play a critical role in efficiently transferring electrons from OM *c*-Cyts to external solid EAs (Marsili et al., 2008). We therefore moved on to test the impacts of the addition of exogeneous flavin on EET efficiency of Δtriple/p*cctA*. After 25 μM RF was added in the anode chamber, the output voltage of Δtriple/p*cctA* rose in 16 h and reached the peak value of 488.8 mV (Figure 7A). In the control experiment with MR-1, the RF addition, similarly, accelerated the voltage generation and elevated the peak voltage by 2.5-fold (313.5 mV vs. 125.1 mV) (Figure 7A). Therefore, to estimate the performance of MFC, this observation was supported by results of the polarization curves and maximum power density measurements (Figure 7B). Compared with the flavin-free control, addition of RF substantially reduced the dropping slope of polarization curves and increased maximum power density to 702.3 mW/m^2^, which was ∼5.8 times higher than that of the WT. Clearly, the presence of flavins significantly further improves EET efficiency.

We further validated the EET efficiency of the Δtriple/p*cctA* strain w/o flavin by assessing rates of MO reduction. Consistently, MO degradation process by the Δtriple/p*cctA* strain was more rapid than that by the WT (Figure 7C). Within 2 h, the engineered strain almost decolorized MO completely, contrasting that the large portion of MO that remained with the control strain was 38%. Expectedly, Δtriple/p*cctA* strain with addition of flavin exhibited a further elevated reduction rate, decolorizing MO completely within 1.5 h. These data collectively indicate that the impacts of the electron shuttle flavin-mediated electron transfer are additive to the optimization of the periplasmic *c*-Cyt redox network.

## Discussion

The ultimate protein basis for conductivity of microbial EET mediated by both direct contact and indirect electron shuttling *via* flavin is *c*-Cyts (Shi et al., 2016; Light et al., 2018; Wang F. et al., 2019; Edwards et al., 2020). In *S. oneidensis* and *Geobacter sulfurreducens*, the two best studied model bacteria for EET, a large number of *c*-Cyts are encoded but only a few have been fully understood in their physiological functions and involvement in EET (Wang Q. et al., 2019). To improve EET efficiency, attempts have been made to elevate the *c*-Cyt content in *S. oneidensis*, which are commonly achieved by increased biosynthesis of cyclic adenosine 3′,5′-monophosphate (cAMP). The cAMP-Crp system serves as the master regulator for transcription of many *c*-Cyt genes, including those critically involved in EET, such as the Mtr system (Saffarini et al., 2003; Charania et al., 2009; Kasai et al., 2019). As cAMP acts as an activator, respiration of a variety of EAs, including those relying on EET, is conceivably compromised by inactivation and low activity, resulting from Crp depletion and lowered cAMP levels, respectively (Saffarini et al., 2003; Charania et al., 2009; Fu et al., 2013; Zhou et al., 2013). Intriguingly, most of the respiratory processes are also negatively affected by Crp overactivation resulting from elevated cAMP concentrations (Jin et al., 2016a; Yin et al., 2016). These observations suggest that changes in cAMP homeostasis impact the physiology of *S. oneidensis* more profoundly than previously anticipated, as reported before (Gao et al., 2010, 2017; Dong et al., 2012; Fu et al., 2013; Zhou et al., 2013).

In this study, we attempted to increase the *c*-Cyt content by manipulating the activity of *c*-Cyt maturation system in *S. oneidensis* MR-1. Through CcmFGH overproduction (San Francisco et al., 2014), more *c*-Cyts can be matured, leading to an increased total *c*-Cyt content in *S. oneidensis*. Although this operation presumably upregulates the production of all *c*-Cyts proportionally, the performance of MFC of *S. oneidensis* is not improved, but unexpectedly impaired. This phenomenon can be readily explained by that certain *c*-Cyts, when present in excess, may be harmful to the EET efficiency of *S. oneidensis*. Indeed, it has been found that in the absence of a few respiratory pathways by removing terminal reductases, including NrfA, CcpA, and NapA, overproduced CctA leads to a 1.7-fold increased ferric iron reduction rate and a 23% higher current generation (Delgado et al., 2019). Given that the approach is effective but the improvement is relatively limited, we reasoned that further optimization of the *c*-Cyt profile should be a feasible strategy to improve EET efficiency.

To identify which *c*-Cyts in excess compromise EET efficiency, each of the “small” periplasmic *c*-Cyts, which primarily function as electron carriers predicted to be capable of shuttling electrons between quinol dehydrogenases and the Mtr system, was expressed in the WT and assayed for their influence on MO reduction (Figure 1). While excessive CctA expectedly shows positive impacts on EET, at least three of such *c*-Cyts, FccA, NapB, and TsdB, when produced at elevated levels, impair EET. By assessing performance of MFC incubated with strains overproducing these proteins, the regulation of these four *c*-Cyts on EET efficiency was validated.

Both FccA and NapB have been extensively studied before, and their effects on EET can be deduced from the current understandings. As the only functioning fumarate reductase (Leys et al., 1999), FccA is an abundant *c*-Cyt also able to act as an electron carrier and deliver electrons from CymA to the Mtr system (Schuetz et al., 2009; Fonseca et al., 2013). Evidence has been presented to suggest that the redox state of FccA determines its function (Paquete et al., 2014). FccA prefers to transferring electrons from CymA to the OM complexes at low electron flux. When the electrons generated by metabolism are sufficient, FccA will be completely reduced to play the role of fumarate reductase (Paquete et al., 2014). Moreover, it has been proposed that the redox state of FccA plays a key role in the interaction between FccA and CymA; when in excess, FccA alters the direction of electron transfer in the CymA-FccA complex, leading to reduced efficiency in all CymA-dependent EET routes (McMillan et al., 2013; Jin et al., 2016a). NapB, a small subunit of nitrate reductase, is a promiscuous electron shuttle protein that can pass electrons to a variety of EAs (Gao et al., 2009; Jin et al., 2016b). Conceivably, when excessive, NapB dissipates electrons obtained from CymA, leading to a decrease in electrons to the Mtr system. In the case of TsdB, this is a first report to uncover a role associated with this protein although the *in silico* analysis suggests that it may catalyze thiosulfate oxidation (Kurth et al., 2016).

The finding that FccA, NapB, and TsdB in overproduction negatively influence EET gains support from the mutational analysis. The loss of each of them, but not any of other *c*-Cyts under test, improves MFC performance. Importantly, these effects are additive, allowing us to construct a most EET-efficient mutant by removing all of them. It has been proposed that the reduced complexity of electron transferring proteins in the periplasm could lead to acceleration of electron transfer (Delgado et al., 2019). Apparently, the data presented there resonate with the proposal. The removal of all of FccA, NapB, and TsdB, which may differ from one another in the mechanisms through which they interfere with electron transfer, the complexity of the periplasmic *c*-Cyt network reduces, leading to the more robust electron flow to the Mtr system. Unlike FccA, NapB, and TsdB, CctA is a unique electron carrier *c*-Cyt capable of transferring electrons from CymA to the Mtr system in an organized and efficient way because it is able to use the same heme to control the entering and leaving of electron (Fonseca et al., 2009; Alves et al., 2015).

By overproducing CctA in the strain devoid of FccA, NapB, and TsdB, we created an engineered strain to reach a peak voltage of 313.5 mV, substantially superior to the WT by nearly three times (125.1 mV). In MFC, this strain could achieve the highest power density output in MFCs (436.5 mW/m^2^), which was up to ∼3.62-fold compared with that of the WT *S. oneidensis*. The improvement appears to exceed that observed in a previous report (Delgado et al., 2019) substantially. However, caution should be taken when comparing these two studies directly because two distinct systems were employed for the assessment: MFC in this study and working electrodes to a specific potential in the other. The EET efficiency of this engineered strain can be further elevated by exogenous addition of RF, up to 50% in overall MFC performance; the maximum output power density reached 702.3 W/m^2^, which was ∼5.8-fold higher than that of the WT. As a small electron shuttle molecule, RF mediates electron transfer from the Mtr complex to electrode, in addition to the direct control (Marsili et al., 2008). The increase in MFC performance by RF addition implies that electron transfer from the Mtr complex to electrode by direct contact is not sufficiently robust to release electrons to extracellular EAs. Given the substantially improving effect of RF addition, a promising strategy for further improvement of EET would be to increase the yield of flavins generated endogenously, which is currently another bottleneck in the engineered strains (Min et al., 2017).

It could be noticed that the coulomb efficiencies obtained in this and early studies (Li et al., 2018) are low, implying the presence of other EAs. Possible candidates would be small redox-active molecules generated during cellular metabolism, such as fumarate, cysteine, and even inorganic sulfur species and nitrogen species (Flynn et al., 2014; Guo et al., 2019). In addition, the output voltage peak of our terminal engineered strain is lagging behind the WT. We do not know the reason underlying the phenomenon yet. The strains with higher EET efficiency, especially Δtriple/p*cctA*, have biofilm growth on the anode slower than the WT. Given that the peak timing coincides with biofilm development, we propose that it is associated with biofilm formation because electroactive biofilm is an important factor in determining output voltage of MFCs (Borole et al., 2011). Biofilm could help establish the contact between cells and the carbon cloth anode and absorb flavins to facilitate direct contacted-based electron transfer and the shuttle-mediated EET (Lin et al., 2018). In addition, EET components are linked to cell surface polarizability, on which the effects of *c*-Cyts vary (Wang Q. et al., 2019). Hence, we envision that rapid electron release in these engineered strains would generate a signal that slows production of extracellular polymeric substance, which not only determines biofilm formation rates but also modulates cell surface polarizability (Wang Q. et al., 2019). Efforts to test this notion are under way.

## Data Availability Statement

The original contributions presented in the study are included in the article/Supplementary Material, further inquiries can be directed to the corresponding authors.

## Author Contributions

HG and SC conceived the idea and designed the project. WS, ZL, and QY carried out the experiments. WS and HG analyzed the data and wrote the manuscript. All authors reviewed the manuscript.

## Conflict of Interest

The authors declare that the research was conducted in the absence of any commercial or financial relationships that could be construed as a potential conflict of interest.

## Publisher’s Note

All claims expressed in this article are solely those of the authors and do not necessarily represent those of their affiliated organizations, or those of the publisher, the editors and the reviewers. Any product that may be evaluated in this article, or claim that may be made by its manufacturer, is not guaranteed or endorsed by the publisher.
