# Supplementary material for: Promoting Extracellular Electron Transfer of Shewanella oneidensis MR-1 by Optimizing the Periplasmic Cytochrome c Network
Source: Front Microbiol. 2021 Oct 5;12:727709. doi: 10.3389/fmicb.2021.727709 (PMC8524038; doi:10.3389/fmicb.2021.727709)
Supplement: Supplementary file 1 [file Presentation_1.pdf]

Supplemental information

Table S1 periplasmic *c*-Cyts of MR-1 investigated in this study

Protein	NO. of heme	Molecular weight (kDa)	Predicted Function
SO3300	4	13.9	flavocytochrome *c* heme submit
SO3056	4	14.2	flavocytochrome *c* heme submit
SO1413	4	13.3	flavocytochrome *c* flavin subunit
SO4666	2	21.9	Important in pellicle formation
SO4485	2	20.4	diheme cytochrome *c*
SO4048 (TsdB)	2	21.9	Thiosulfate oxidation
SO4484	1	15.9	Possible monoxygenase
SO4142	1	12.0	Unknown
SO3420	1	16.3	Unknown
SO0717	1	12.1	Sulfite dehydrogenase subunit
SO0714	1	11.3	
ScyA	2	10.4	involved in cbb_3 oxidase activity
CctA (STC)	4	12.3	Electron carrier
NapB	2	15.4	Subunit of nitrate reductase
FccA	4	62.5	Fumarate reductase

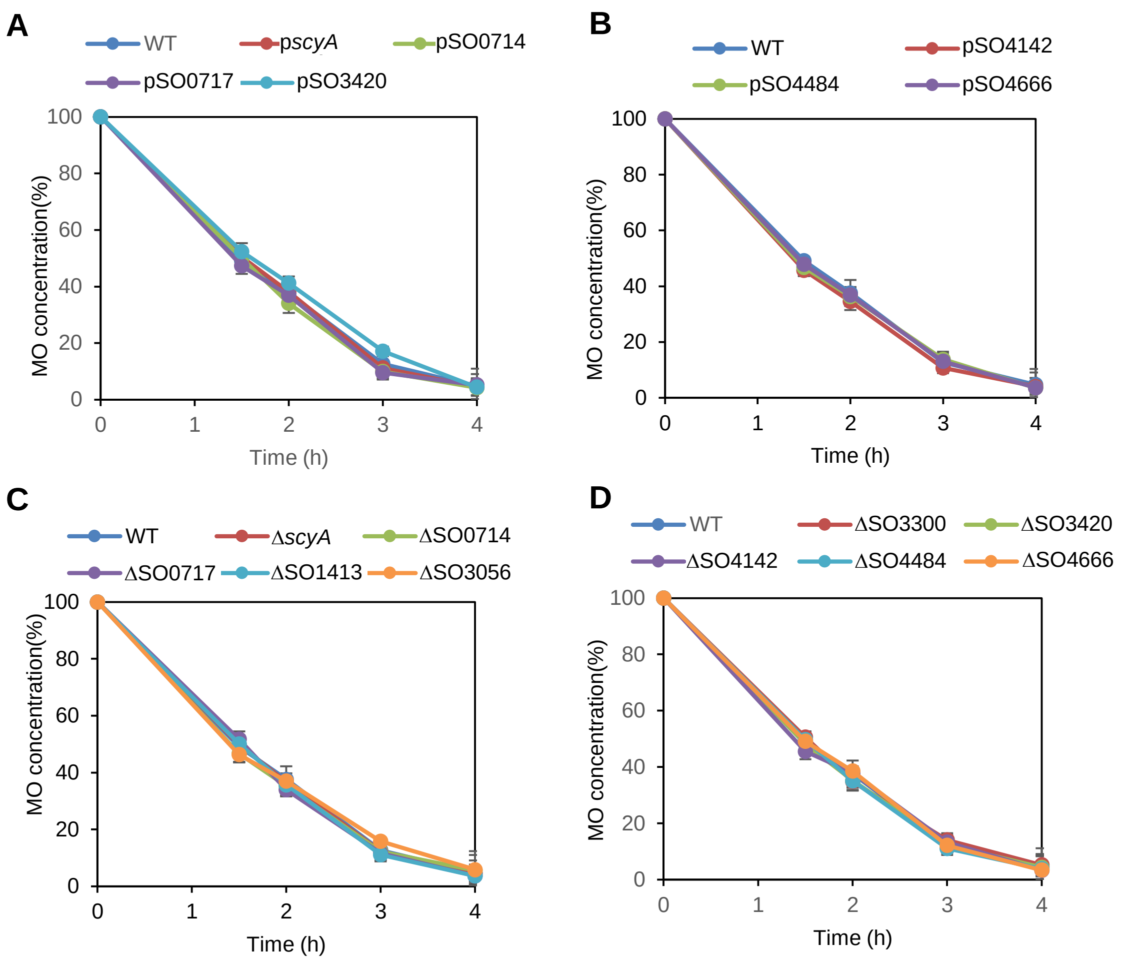

FIGURE S1. The periplasmic *c*-Cyts in overabundance interfere with EET efficiency. (A) (B) MO degradation rates of the indicated strains grown with 0.5 mM IPTG. (C) (D) MO degradation rate of periplasmic *c*-Cyt single mutants grown with 0.5 mM IPTG. Experiments were performed in at least three independent operations, and data are presented by the means ± SD. The differences in values at each time point between WT and each of mutants are insignificant.

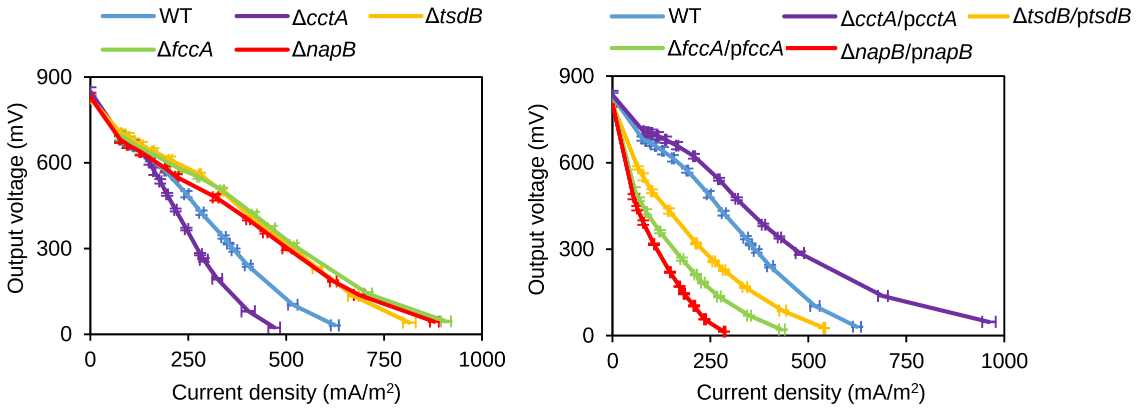

FIGURE S2 Bioelectrochemical characterization in dual-chamber MFCs of the periplasmic *c*-Cyt mutants and their complementary strains induced by 0.5 mM IPTG. Polarization curves determined by measuring the stable output voltage generated across various external resistances. Experiments were performed in at least three independent operations, and data are presented by the means ± SD.

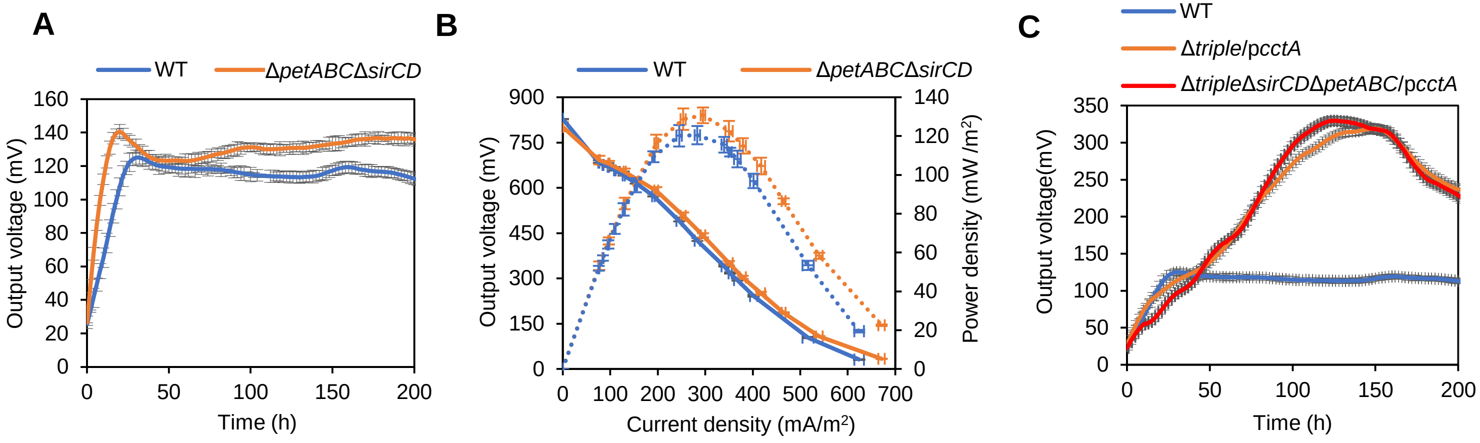

FIGURE S3 Impacts of quinol oxidases other than CymA on MFC performance. (A) Output voltage of MFCs incubated with Δ*petABC*Δ*sirCD* and the control strain across the 2000 Ω external resistor. (B) Polarization curves (solid lines) and power density curves (dash lines) determined by measuring the stable output voltage generated across various external resistances. Reproducibility was examined with at least three independent operations. (C) Output voltage of MFCs incubated with Δ*triple*Δ*sirCD*Δ*petABC*/p*cctA* and the control strain across the 2000 Ω external resistor. Experiments were performed in at least three independent operations, and data are presented by the means ± SD. In (A) and (B), the differences in the peak values between WT and the mutant under test were < 0.05. In (C), the difference in the peak values between the mutant strains expressing *cctA* and not was insignificant.
